# *N*-((1*H*-Pyrrol-2-yl)methylene)-6-methoxypyridin-3-amine and Its Co(II) and Cu(II) Complexes as Antimicrobial Agents: Chemical Preparation, In Vitro Antimicrobial Evaluation, In Silico Analysis and Computational and Theoretical Chemistry Investigations

**DOI:** 10.3390/molecules27041436

**Published:** 2022-02-21

**Authors:** Vinusha H. Mariwamy, Shiva Prasad Kollur, Bindya Shivananda, Muneera Begum, Chandan Shivamallu, Chandan Dharmashekara, Sushma Pradeep, Anisha S. Jain, Shashanka K. Prasad, Asad Syed, Abdallah M. Elgorban, Salim Al-Rejaie, Joaquín Ortega-Castro, Juan Frau, Norma Flores-Holguín, Daniel Glossman-Mitnik

**Affiliations:** 1 Department of Chemistry, Sri Jayachamarajendra College of Enegineering, JSS Science and Technology University, Mysuru 570 006, Karnataka, India; vinushahm@gmail.com (V.H.M.); bindyas@jsstuniv.in (B.S.); begum@sjr.ac.in (M.B.); 2 School of Agriculture, Geography, Environment, Ocean and Natural Sciences (SAGEONS), Laucala Campus, The University of the South Pacific, Suva, Fiji; 3 Department of Sciences, Amrita School of Arts and Sciences, Mysuru Campus, Amrita Vishwa Vidyapeetham, Mysore 570 026, Karnataka, India; 4 Department of Biotechnology and Bioinformatics, School of Life Sciences, JSS Academy of Higher Education and Research, Mysuru 570 026, Karnataka, India; chandand@jssuni.edu.in (C.D.); sushmap@jssuni.edu.in (S.P.); anishasjain@jssuni.edu.in (A.S.J.); shashankaprasad@jssuni.edu.in (S.K.P.); 5 Department of Botany and Microbiology, College of Science, King Saud University, P.O. Box 2455, Riyadh 11451, Saudi Arabia; assyed@ksu.edu.sa (A.S.); aelgorban@ksu.edu.sa (A.M.E.); 6 Department of Pharmacology and Toxicology, College of Science, King Saud University, P.O. Box 2455, Riyadh 11451, Saudi Arabia; rejaie@ksu.edu.sa; 7 Departament de Química, Universitat de les Illes Balears, E-07122 Palma de Mallorca, Spain; joaquin.castro@uib.es (J.O.-C.); juan.frau@uib.es (J.F.); 8 Laboratorio Virtual NANOCOSMOS, Departamento de Medio Ambiente y Energía, Centro de Investigación en Materiales Avanzados, Chihuahua 31136, Mexico; norma.flores@cimav.edu.mx

**Keywords:** Schiff base, antimicrobial activity, molecular docking, spectroscopic techniques, Conceptual DFT, biological scores, ADMET

## Abstract

Researchers are interested in Schiff bases and their metal complexes because they offer a wide range of applications. The chemistry of Schiff bases of heterocompounds has got a lot of attention because of the metal’s ability to coordinate with Schiff base ligands. In the current study, a new bidentate Schiff base ligand, *N*-((1*H*-pyrrol-2-yl)methylene)-6-methoxypyridin-3-amine (MPM) has been synthesized by condensing 6-methoxypyridine-3-amine with pyrrole-2-carbaldehyde. Further, MPM is used to prepare Cu(II) and Co(II) metal complexes. Analytical and spectroscopic techniques are used for the structural elucidation of the synthesized compounds. Both MPM and its metal complexes were screened against *Escherichia coli*, *Bacillus subtilis*, *Staphylococcus aureus* and *Klebsiella pneumoniae* species for antimicrobial studies. Furthermore, these compounds were subjected to in silico studies against bacterial proteins to comprehend their best non-bonded interactions. The results confirmed that the Schiff base ligand show considerably higher binding affinity with good hydrogen bonding and hydrophobic interactions against various tested microbial species. These results were complemented with a report of the Conceptual DFT global reactivity descriptors of the studied compounds together with their biological scores and their ADMET computed parameters.

## 1. Introduction

Schiff base is an amine ligand synthesized through a condensation reaction between primary amine group and carbonyl group of ketone or aldehyde forming an azomethine group (-C=N-) [1]. Schiff base ligands with heterocyclic structure containing Nitrogen, Sulphur or Oxygen, exhibits a broad application in various fields like medicine, corrosion inhibitors, catalytic activity, biological activity, etc. [2,3,4,5,6,7]. As Schiff base organic compounds exhibits a wide spectrum of biological activities such as antiinflammatory drugs [8,9,10], antimicrobial [11,12,13,14], anticonvulsant [15], tuberculosis [16], anticancer [17,18], antioxidants [19] and antihelmintic [20], they are known as biologically high potent compounds. The Schiff base ligand acting as bidentate or tridentate can easily form stable complexes with transition metals. The attention of the researchers is drawn towards the Schiff base metal complexes in coordination chemistry because of their enhanced bio relevant and pharmacological activities such as DNA/protein targeting, anticancer, antioxidant activity [21,22,23]. In comparison to other transition metal complexes, the Schiff base-copper(II) complexes are said to play a crucial role in nucleic acids chemistry because of their stable structures and relevance to biological proteins. Antibacterial, antifungal, antiviral, antiproliferative and anticancer action can be found in heterocyclic Schiff base Co(II) complexes.

They are also used as catalyst in many organic reactions [24]. Thus, extensive growth of new chemotherapeutic Schiff base ligands and their metal complexes play a vital role in Bio-inorganic and Medicinal chemistry [25]. In recent years, much of scope and focus is given towards the study of biologically potent compounds to meet the health issues in society [26]. Our research work is aimed to design, synthesize and characterize the new pyridine derived Schiff base ligand and its transition metal complexes. The work is extended to study the biological properties of ligand and its metal complexes synthesized by evaluating their biological activity on different assay systems.

## 2. Experimental

### 2.1. Materials and Methods

All the chemicals and solvents required were purchased from Sigma and used without further purification. The infrared spectra were recorded using a Perkin Elmer FT-IR type 1650 spectrophotometer in the region within 4000–400 cm${}^{-1}$ considering KBr pellets. NMR spectra were recorded in DMSO-d6 as a solvent against tetramethylsilane as an internal standard on a Varian 300 MHz. An Agilent technologies (HP) 5973 mass spectrometer was used to perform mass spectrometric analyses at a 70 eV ionization potential. Spectrophotometric measurements were performed on aUV1800 spectrophotometer (Shimadzu). The thermogravimetric analysis (TGA) was performed using a Universal TGA Q50 instrument at a heating rate of 2 °C/min within a range of 30 and 1000 °C.

### 2.2. Biology

All the patients’ samples like sputum, pus/exudates, blood, urine etc., are collected from Department of Microbiology, JSS Hospital, Mysuru and samples are screened and isolated with bacteria such as *Bacillus subtilius*, *Staphylococcus aureus*, *Escherichia coli* and *Klebsiella pneumoniae*. The isolated four colonies were sub-cultured on Luria–Bertani (LB) broth media, a nutritionally rich media specially designed for the growth of bacteria. After 24 h of incubation, bacterial colonies were settled at the bottom of the LB broth tubes. Bacterial colonies were examined microscopically and further processed to antibiotic susceptibility test [27].

### 2.3. Synthesis of N-((1H-Pyrrol-2-yl)methylene)-6-methoxypyridin-3-amine (MPM)

**Scheme 1:**
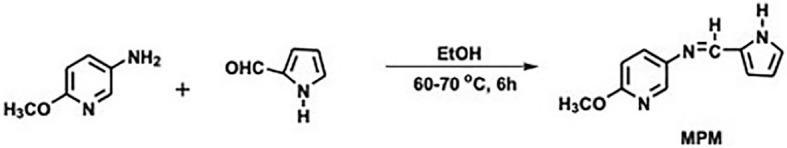
Synthesis of Schiff base ligand, **MPM**.

The MPM is prepared according to Scheme 1. An equimolar mixture of 6-methoxy-pyridine-3-amine dissolved in 20 mL of ethanol and pyrrole-2-carbaldehyde in 10 mL of ethanol was refluxed for 6 h at 65 °C with catalytic amount of glacial acetic acid. The completion of the reaction was assessed through TLC. The mixture was kept for evaporation at room temperature. Brown colored solid obtain was washed with ethanol, dried and preserved to obtain the desired product.

### 2.4. Synthesis of Schiff Base Transition Metal Complexes (MPM-Cu(II) and MPM-Co(II))

**Figure 1:**
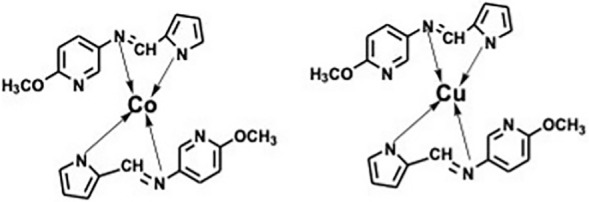
Proposed structure for the MPM-Co (II) (**left**) and MPM-Cu (II) (**right**) complexes.

20 mL of warm ethanolic solution of 0.5 mM, 0.1 g ligand (MPM) and 20 mL, 0.25 mM metal chloride salt [0.042 g CuCl_2_·H_2_O and 0.06 g CoCl_2_·6H_2_O dissolved in ethanol were mixed together in 2:1 ratio. The mixture was stirred well for 30 min with few drops of sodium acetate solution. The precipitate of Schiff base metal complexes obtained were filtered, washed with distilled water and dried under vacuum (Figure 1 and Table 1).

### 2.5. Antimicrobial Activity

In the present investigation, the in vitro antimicrobial screening of newly synthesized Schiff base and its complexes was performed using agar disk diffusion assay considering two Gram positive bacteria (*Bacillus subtilis* and *Staphylococcus aureus*) and two Gram negative bacteria (*Escherichia coli* and *Klebsiella pneumoniae*), where Chloramphenicol was used as control to investigate the potency of compounds being studied under same conditions, while these bacterial cultures were incubated for about 24 h and were spread on Muller–Hinton agar plate. A sterile disk of 6mm Whatman paper was saturated with 10 μL of 8-hydroxyquinoline derivatives solution. After 1 h of diffusion, the culture plates were again incubated at 37 °C for 24 h and the obtained zone of inhibition were measure and compared with Chloramphenicol reference discs [28,29]. Activity study was carried out by taking the compounds in different concentrations viz., 0.25, 0.5, 0.75 and 1.0 mg/mL.

### 2.6. Bacterial Growth Curve

The bacterial growth curve was conducted as described by Venkata et al. [30] with slight modification. The assay was performed using sterilized 96 well microplates. The plate lid was covered with 4 mL of 0.05% Triton X-100 dissolved in 20% ethanol and was incubated at 15 s at room temperature to avoid water reduction on the microplate. The plate was dried before the measurements. To evaluate the bacterial growth curve of *E. coli*, *B. subtilis*, *K. pneumoniae* and *S. aureus* using selected bacterial culture, 200 μL of selected bacterial culture were added to the microplate wells. The plates were placed in a microplate reader to examine the OD (optical density) of the bacterial culture at 600 nm. The obtained microplate wells were corrected by multiplying the photometer pre-calibrated factor 2.39. The plate was incubated in a rotator incubator at 37 °C and 355 rpm. The optical density of the individual well was measured for every 10 min during 16 h.

### 2.7. MTT Assay

Using an appropriate medium containing 10% FBS, the monolayer culture was trypsinized and modified to 5.0 × 105 cells/mL. A total of 100 μL of diluted cell suspension (50,000 cells/well) was added to each well in the microtiter plate. Past 24 h, a monolayer had developed, the supernatant was swept off, the obtained monolayer was washed with media and 100 μL of various test drug doses were applied to the monolayer in microtiter plates. Further incubation of the plates was carried out for 24 h at 37 °C in a 5% CO${}_{2}$ environment. The test solutions in the wells were removed after incubation and to each well 100 μL MTT was added. In a 5% CO${}_{2}$ environment and at 37 °C, the plates were incubated for 4 h. The supernatant was withdrawn and 100 μL of DMSO was added to the plates, which were gently agitated to dissolve the formazan that had formed. At a wavelength of 590 nm, the absorbance was measured using a microplate reader. The growth inhibition percentage was estimated using the below-mentioned formula and the dose-response curves were used to estimate the test drug concentration required to inhibit cell growth by 50% (IC50) values [31]. The following formula was used to compute the percentage of inhibition:

$$\left(\%\right)\mathrm{Inhibition}=\left[\frac{\mathrm{OD}\mathrm{of}\mathrm{Control}-\mathrm{OD}\mathrm{of}\mathrm{Sample}}{\mathrm{OD}\mathrm{of}\mathrm{Control}}\right]\times 100$$

### 2.8. In Silico Analysis

#### 2.8.1. Ligand Preparation

**Figure 2:**
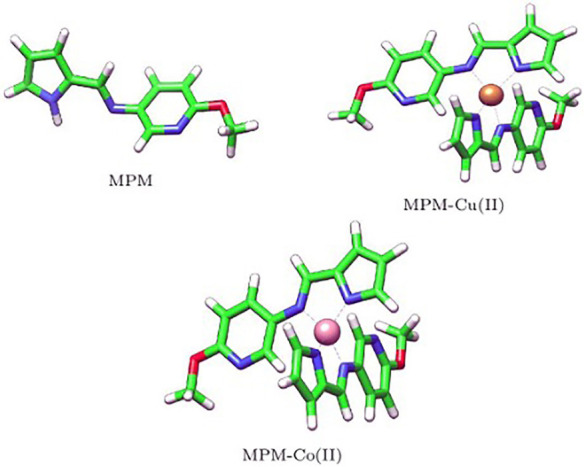
Structures of MPM, MPM-Cu(II) and MPM-Co(II) drawn from ChemSketch software and visualized using Chimera software.

In order to carry out the Molecular Docking process, the structures of the synthesized ligand and its Cu(II) and Co(II) complexes were drawn using the ChemSketch tool of the ACDLABS 11.0 chemical drawing package. The structure files generated from this tool were further converted into 3-dimensional structure files using the OpenBabel GUI by generating the 3D coordinates and by adding hydrogen atoms explicitly. The geometry of the structures was cleaned using the Argus Lab software and energy minimization of all the synthesized ligands was carried out using the visualization tool, Chimera, to remove clashes among atoms. The ligand and its Cu(II) and Co(II) complex structures were now ready to be docked into the active sites of the receptor as shown in the Figure 2.

#### 2.8.2. Protein Preparation

**Figure 3:**
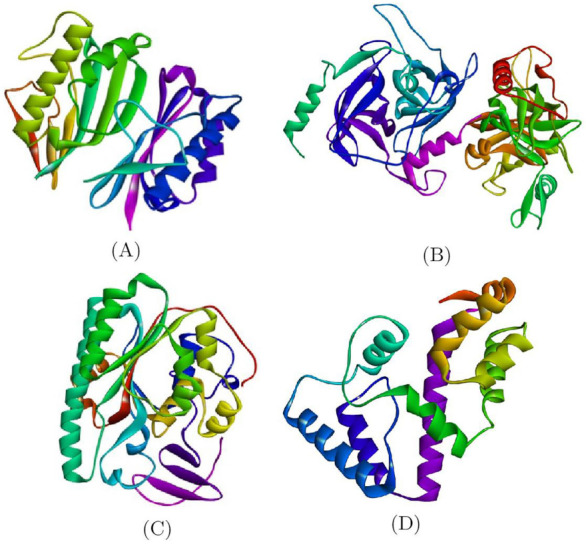
Crystal Structures of bacterial proteins: (**A**) *Bacillus subtilis*, (**B**) *Staphylococcus aureus*, (**C**) *Escherichia coli* and (**D**) *Klebsiella pneumoniae*.

In this study all the compounds were checked for the antimicrobial activity against Gram-negative and Gram-positive bacteria. These bacterial protein crystal structures of *Bacillus subtilis*, *Staphylococcus aureus*, *Escherichia coli* and *Klebsiella pneumoniae* [32,33,34] were downloaded from the Protein Data Bank (PDB) and are shown in Figure 3. These proteins were considered as the macromolecules for the further Molecular Docking studies with selected ligands. The targeted binding site residues for each protein were downloaded from the CastP Web server.

### 2.9. Molecular Docking Studies

Molecular docking was performed on the bacterial proteins against synthesized compounds to analyze the non-bonded interactions and binding affinity of the docked compounds using PyRx v.0.8, a virtual screening tool [35]. The prepared proteins and ligands were uploaded to the software as macromolecule and ligand, respectively.

The ligand and its complexes are docked in a grid box around the binding site residues of the target proteins, resulting in the optimal conformation with the lowest binding affinity value (Kcal/mol) [36].

#### Molecular Docking Interaction Analysis

The resulted docked poses of the ligands with the target proteins were further analyzed using PyMol v.2.5 and LIGPLOT+ v.2.2 software. Of the obtained nine poses, one with the lowest binding energy value and high non-bonded interactions was considered for further analysis. The visualizing tools could predict all the non-bonded interactions such as hydrogen bonds, hydrophobic interactions, electrostatic interactions along with their bond lengths [37]. The 2D and 3D images were generated by the software for better analysis of the interactions.

### 2.10. Conceptual DFT Studies

The molecular energies, electronic densities and orbital energies of MPM and its metal complexes were determined using the Kohn-Sham (KS) approach [38,39,40,41] while making use of the Conceptual DFT (CDFT) methodology [42,43,44,45,46,47,48]. The conformers of the compounds investigated in this study were calculated using MarvinView 17.15 from ChemAxon (http://www.chemaxon.com, accessed on 1 July 2021) by performing Molecular Mechanics calculations with the complete MMFF94 force field [49,50,51,52,53]. The next step was the full geometry optimization and frequency calculation using MN12SX/Def2TZVP/H2O model chemistry [54,55,56] and the estimation of the electronic properties and the chemical reactivity descriptors of the studied ligands considering the same model chemistry [54,55,56] on the basis of their optimized molecular structures. This model chemistry is based on the MN12SX screened-exchange density functional [54] and the Def2TZVP basis set [55,56] considering the charge of the compounds as being equal to zero. This determination was performed with the aid of the Gaussian 16 software [57] and the SMD solvation model [58] and owing to the fact that the mentioned model chemistry has been previously proved as verifying the ’Koopmans in DFT’ (KID) procedure [59,60,61,62]. This last step was also required for the verification of the absence of imaginary frequencies as a check for the stability of the optimized structures as being a minimum in the energy landscape (accessed on 1 July 2021).

#### Computational ADMET

During the development of a novel therapeutic drug, it is vital to understand pharmacokinetics, or the fate of a molecule in the body. Individual indices called Absorption, Distribution, Metabolism, Excretion and Toxicity (ADMET) variables are commonly utilized. Computer models are frequently used as an alternative to using experimental methods to establish these parameters. Chemicalize, a software developed by ChemAxon (http://www.chemaxon.com, accessed on 1 July 2021) was considered for this purpose with additional information about the pharmacokinetics parameters and the ADMET properties obtained through admetSAR [63], on the basis of their SMILES notation (http://lmmd.ecust.edu.cn/admetsar2/) (accessed on 1 July 2021). Molinspiration software (https://www.molinspiration.com/) (accessed on 1 July 2021) has been used to calculate various molecular properties and to predict the bioactivity scores for several drug targets of interest for the process of drug discovery.

## 3. Results

### 3.1. Chemistry

**Figure 4:**
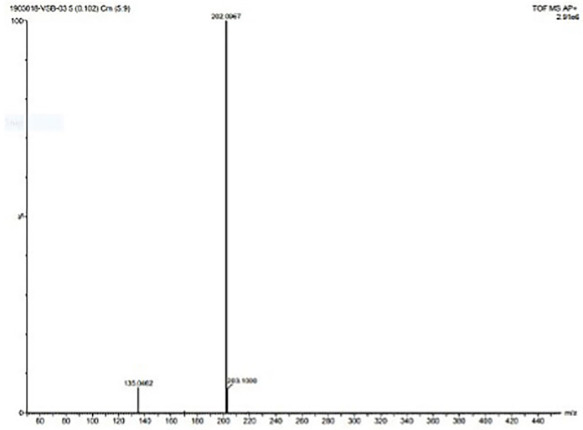
Mass spectrum of MPM.

**Figure 5:**
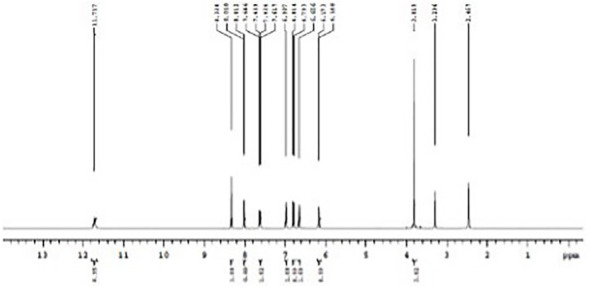
${}^{1}$H NMR spectrum of MPM.

**Figure 6:**
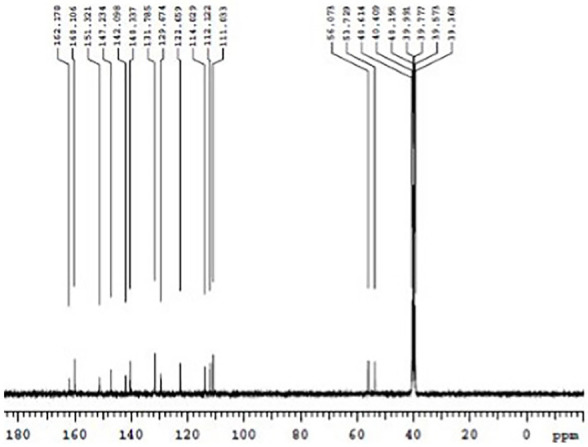
${}^{13}$C NMR spectrum of MPM.

The formation of Schiff base and their metal complexes were verified by using mass spectroscopy. The mass spectrum of ligand was shown in Figure 4. The molecular ion peak at *m*/*z* = 202.09 confirms the stoichiometry of the ligand. Figures S1 and S2 shows the mass spectrum of MPM-Cu(II) and MPM-Co(II) complexes. In the mass spectrum of complexes, the molecular ion peaks at 463.125 and 458.26 confirm the coordination of Cu and Co ions, respectively, with the MPM. Figure 5 shows the ${}^{1}$H NMR spectrum of the ligand. The singlet peak at 8.338 ppm corresponds to imine proton (CH=N-) of MPM ligand. The signals observed in the region between 8.018-6.168 ppm corresponds to aromatic protons of the ligand. The corresponding ${}^{13}$C NMR spectrum of MPM ligand is provided in Figure 6. The signal at 162.176 ppm was attributed to (-CH=N). Methoxy group (-OCH3) carbon of MPM was observed at 53.72 ppm. The peaks of other aromatic ring carbons were observed at 111.03 ppm to 160.10 ppm. These peaks support the formation of MPM ligand [64,65].

**Figure 7:**
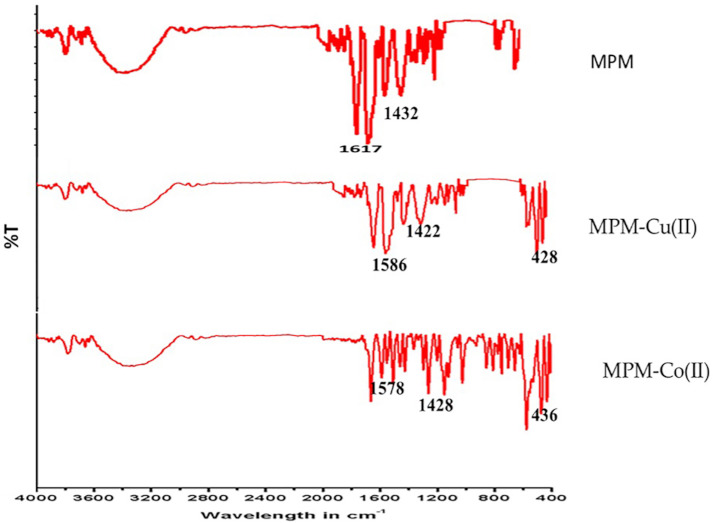
IR spectrum of MPM.

**Figure 8:**
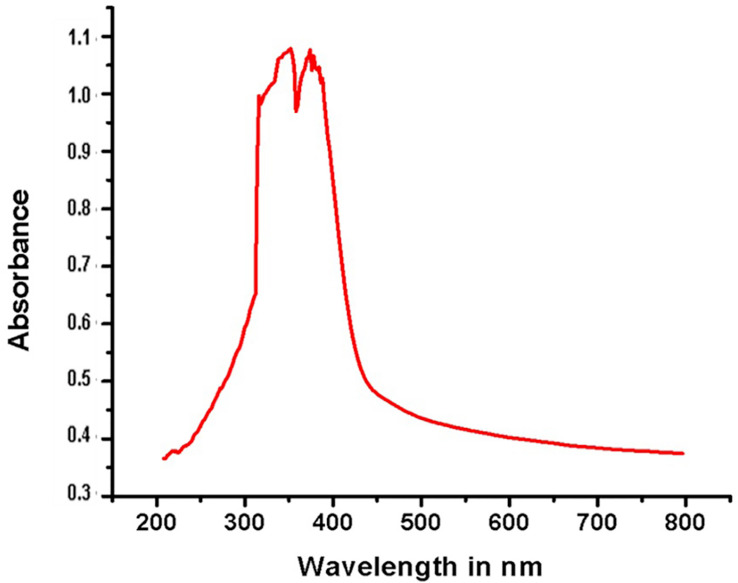
UV-Visible spectrum of MPM.

The formation of the ligand and its complexes were confirmed by IR analysis. FT-IR spectra of the MPM and its metal complexes were compared on the basis of any changes in the bands during the coordination. The comparison of the infrared spectral data of the ligand and its complexes confirmed that complexation occurred, as significant shifts were observed in the bands of the azomethine group ν(CH=N). Figure 7 depicts a sharp band at 1617 cm${-}^{1}$ corresponding to an imine group stretching vibration [66]. In the IR spectra of the complexes (Figures S3 and S4), the appearance of new bands was considered as a sign of coordination between the metal ions [67,68]. The UV-Visible spectrum of MPM ligand (Figure 8) and their Cu (II) and Co (II) complexes was depicted in Figures S5 and S6. Two bands were absorbed at 351 and 372 nm corresponding to π→$\pi^{*}$ transition of the heterocyclic moiety and n →$\pi^{*}$ transition of the azomethine group of the ligand, respectively. As a result of the coordination to metal, the π→$\pi^{*}$ and n →$\pi^{*}$ transitions in the metal complexes were displaced to longer wavelengths, confirming the synthesis of the Schiff base metal complexes [69].

The thermal analysis (TG and DTG) evidences the stepwise degradation of complex molecule (Figures S7 and S8). The TGA curves of the metal complexes give three stage decomposition pattern. In MPM-Cu(II) complex, the first degradation stage is noticed in the range of 28.71–470.13 °C with 37.92% weight loss due to the loss of water molecule. The second degradation stage occurs in the range of 470.13–700.19 °C with a weight loss of 57.69% corresponds to the loss of organic moiety present in a molecule and the third degradation stage takes place in the range of 700.19–798.97 °C which is corresponds to the loss of Schiff base ligand leaving behind the residue of 4.248% for Cu(II) metal complex. In MPM-Co(II) complex, the first degradation stage is observed in the range of 26.94–132.33 °C with a weight loss of 12.84%. It is due to the elimination of water molecule. The second degradation stage occurs in the range of 132.33–534.69 °C with a weight loss of 33.78% due to the loss of organic moiety present in a molecule and the third degradation stage takes place in the range of 534.69–770.55 °C with a weight loss 39.71%. This is due to the loss of Schiff base ligand leaving behind the residue of 16.42% for Co(II) metal complexes [70,71,72].

Although single-crystal X-ray diffraction of the Cu(II) and Co(II) complexes could be of interest for a further characterization of these systems, it had been impossible to obtain good single crystals that were suitable for recording the crystal structure.

### 3.2. Antimicrobial Activity

The antimicrobial activity of the Schiff base ligand and its metal complexes was tested against *Bacillus subtilis* and *Staphylococcus aureus*, which are examples of Gram-positive bacteria as well as against *Escherichia coli* and *Klebsiella pneumoniae* which are representative of Gram-negative class. Chloramphenicol was used as a standard antibiotic for comparison of antibacterial activities. All the tested compounds showed a good antibacterial activity at highest concentration in case of Gram-negative bacterial species (*Escherichia coli* and *Klebsiella pneumoniae*), whereas those compounds exhibit less activity against Gram-positive bacteria (*Bacillus subtilis* and *Staphylococcus aureus*). Further, it is observed that the ligand showed better activity than its complexes. By considering these results, the chemically synthesized Schiff base ligand may consider the formulation of novel chemotherapeutic agents (Table 2).

### 3.3. Bacterial Growth Curve

**Figure 9:**
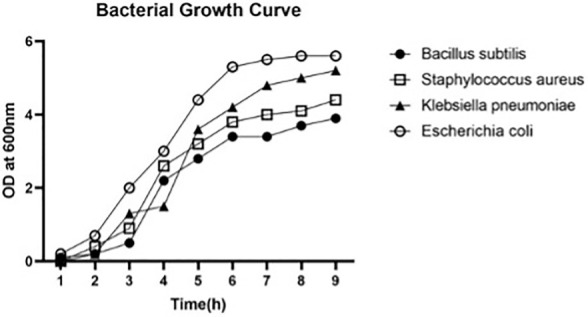
Bacterials growth curve of *B. subtilus*, *S. aureus*, *E. coli* and *K. pneumoniae*.

The bacterial growth curve of *K. pneumoniae*, *S. aureus*, *B. subtili* and *E. coli* was measured based on their turbidity using a microplate reader at 620 nm as shown in Figure 9. All the bacteria grew in the same form but with different turbidity. The cultures were initially measured in the log phase (6–8 h) followed by the stationary phase. The lag phases of *Escherichia coli* and *Klebsiella pneumoniae* were found to be longer than those for *Bacillus subtilis* and *Staphylococcus aureus*.

### 3.4. MTT Assay

**Figure 10:**
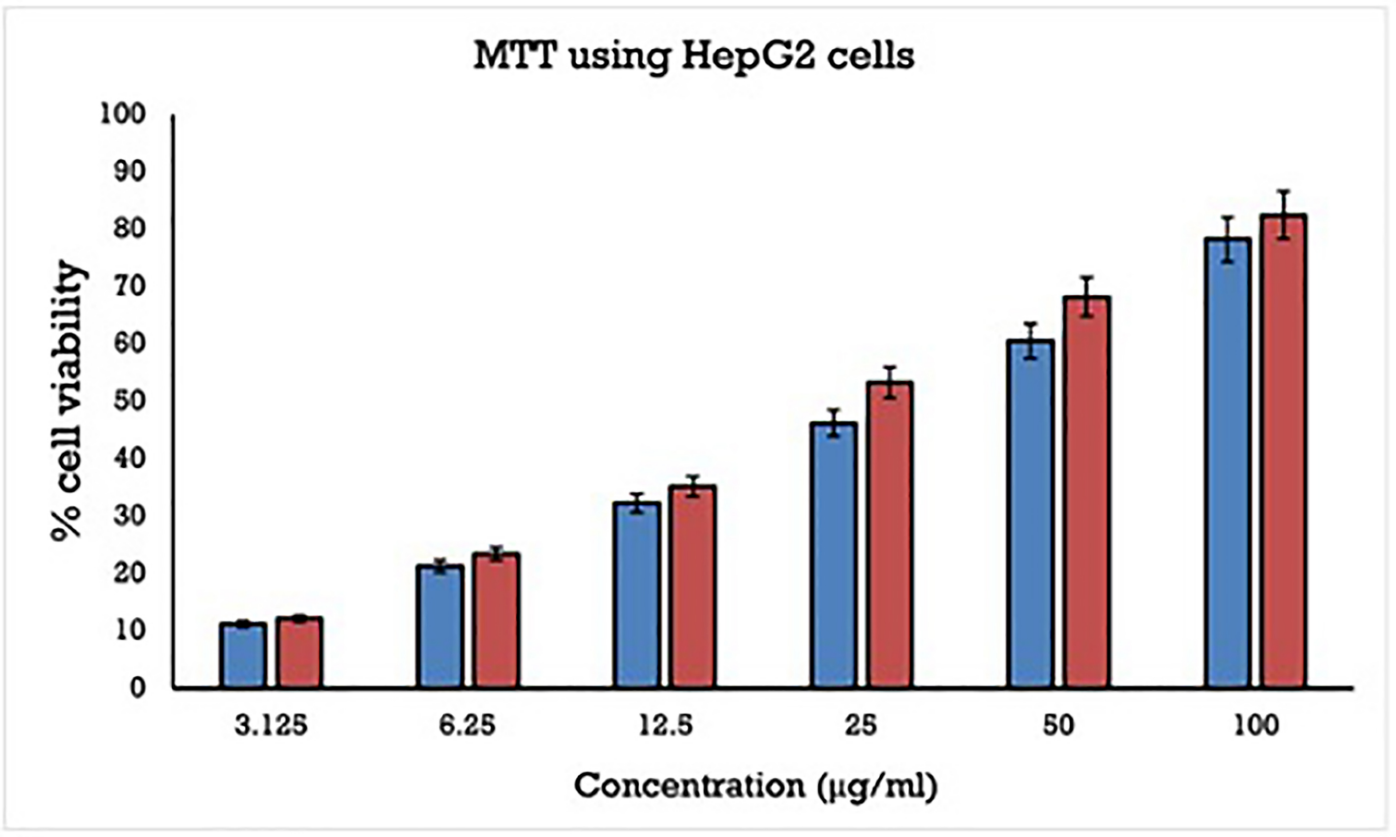
Citotoxicity effect of the synthesized MPM compound and standard drug Doxorubicin against HepG2 cells for about 24 h using MTT assay at different concentrations ranging from 3.125 to 200 μg/mL.

The hepatoprotective potential of the synthesized MPM in HepG2 cells was observed by MTT assay as shown in Figure 10. The percentage reduction of cell viability using synthesized MPM compound were compare with standard drug doxorubicin against HepG2 cells were performed for about 24 h using MTT assay. The MPM compound and a standard drug doxorubicin were treated with different concentration 3.125, 6.25, 12.5, 25, 50 and 100 μg/mL against HepG2 cells lines for about 24 h which shows the significant reduction of cell viability in a concentration-dependent manner. The obtained results confirm that IC50 value of synthesized MPM compound shows 32.52 μM while standard Doxorubicin shows 23.43 μM in HepG2 cells.

### 3.5. Molecular Docking Interactions

**Figure 11:**
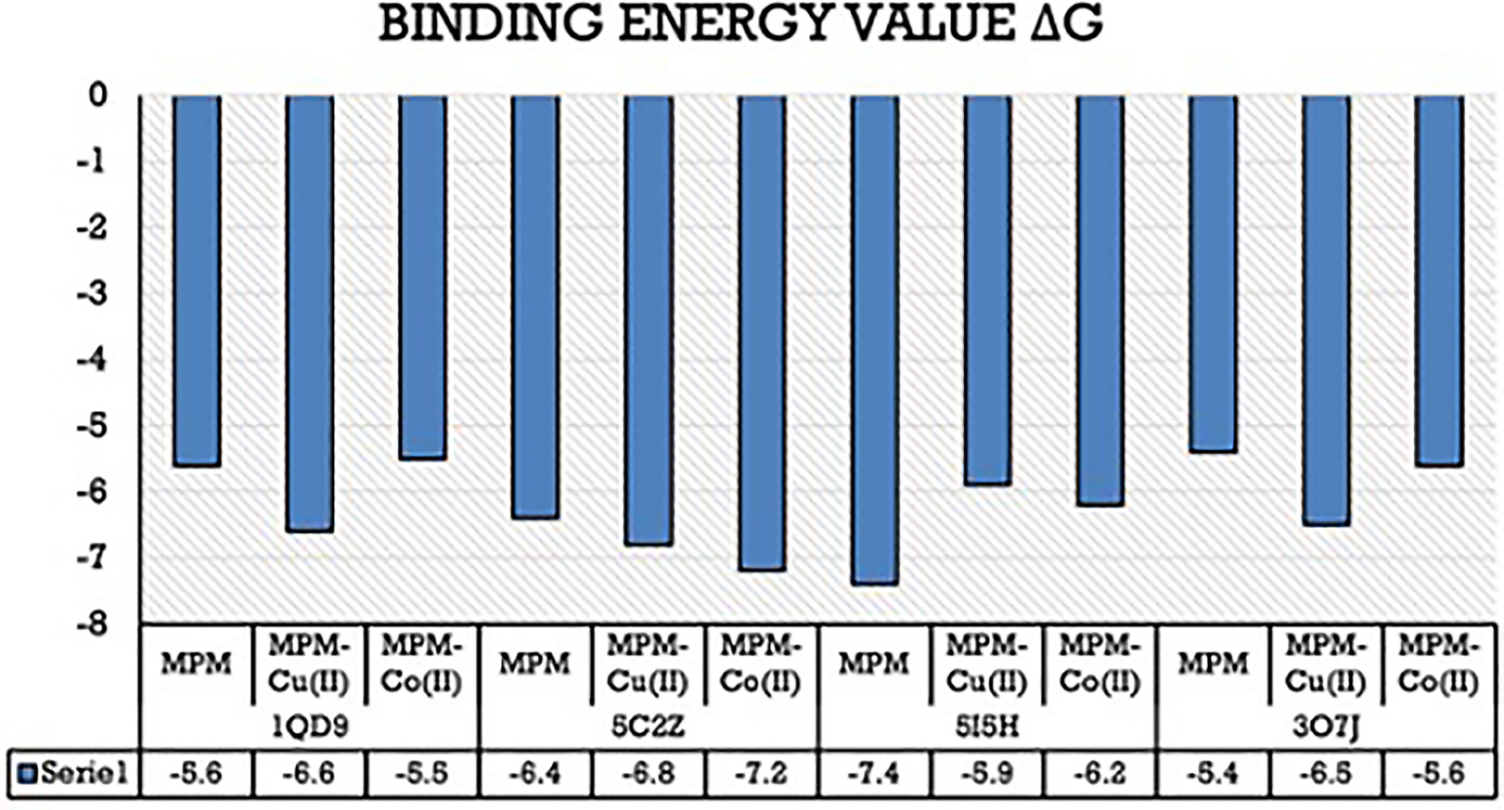
A bar graph depicting the binding affinity values (Kcal/mol) of each ligand docked to the active site of respective proteins.

Post molecular docking, the best-docked poses of all the molecular docked complexes were taken into consideration and the corresponding lowest binding energy values were noted down. The amino acid residues forming the non-bonded interactions between the ligands and the target proteins were determined by the visualization software (Table 3 and Figure 11).

#### 3.5.1. Docking Analysis of *Bacillus subtilus* Protein(1Q29) with Synthesized Compounds

**Figure 12:**
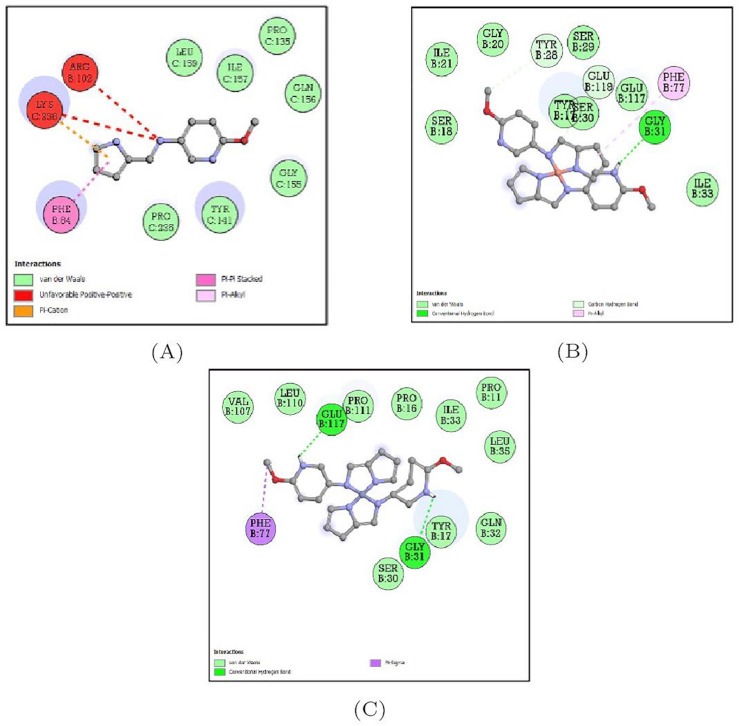
2D representation of the non-bonded interactions between the binding residues of 1QD9 protein with (**A**) MPM, (**B**) MPM-Cu(II) and (**C**) MPM-Co(II).

The docking interactions of 1QD9 target protein with all three compounds are represented in Figure S9. Of the three compounds, this protein bound to the MPM-Cu(II) complex showed the least binding energy (−6.6 Kcal/mol) and a single hydrogen bond with GLY-31 and more van der Waals interactions were observed. The binding energy of the MPM ligand was estimated to be −5.6 Kcal/mol while that of MPM-Co(II) complex was −5.5 Kcal/mol and the amino acid residues forming the bonds are depicted in Table 3 and Figure 12.

#### 3.5.2. Docking Analysis of *Staphylococcus aureus* Protein (5C2Z) with Synthesized Compounds

**Figure 13:**
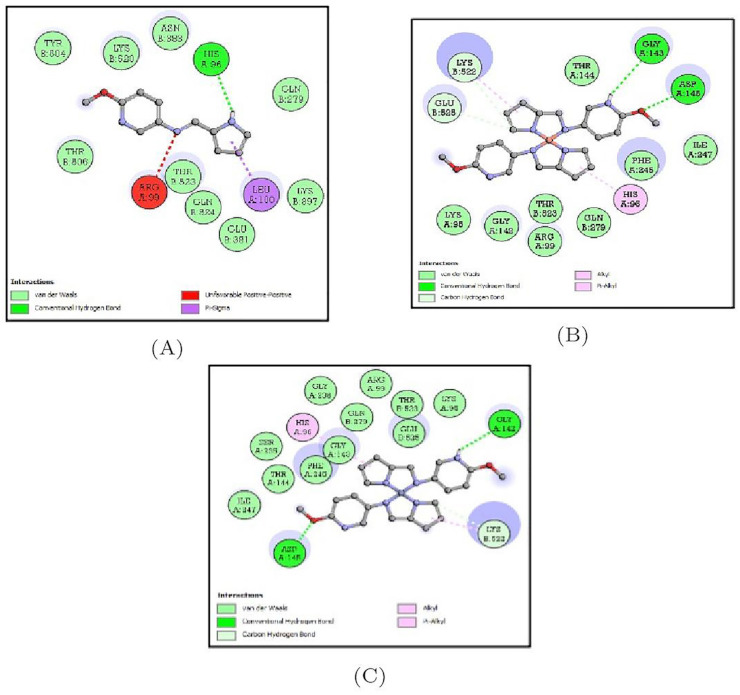
2D representation of the non-bonded interactions between the binding residues of 5C2Z protein with (**A**) MPM, (**B**) MPM-Cu(II) and (**C**) MPM-Co(II).

The docking interactions of 5C2Z target protein with all three compounds are represented in Figure S10. Of the three compounds, this protein bound to the MPM-Co(II) complex showed the least binding energy (−7.2 Kcal/mol) and two hydrogen bonds with ASP-145, GLY-142 with a high number of residues participating in van der Waals interactions. The binding energy of the MPM ligand was estimated to be −6.4 Kcal/mol while that of the MPM-Co(II)complex was −6.8 Kcal/mol and the amino acid residues forming the bonds are depicted in Table 3 and Figure 13.

#### 3.5.3. Docking Analysis of *Escherichia coli* Protein (5I5H) with Synthesized Compounds

**Figure 14:**
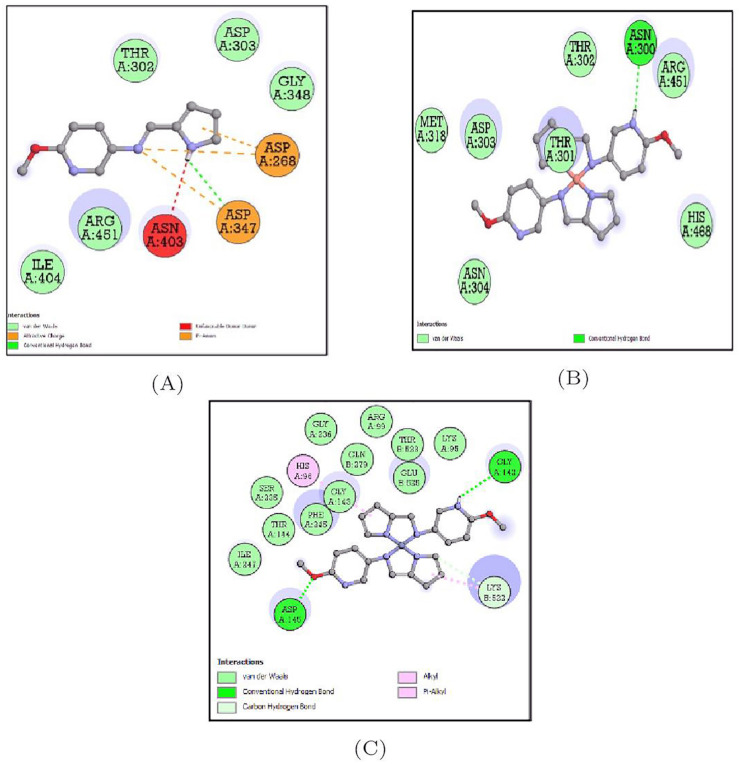
2D representation of the non-bonded interactions between the binding residues of 5I5H protein with (**A**) MPM, (**B**) MPM-Cu(II) and (**C**) MPM-Co(II).

The docking interactions of 5I5H target protein with all three compounds are represented in Figure S11. Of the three compounds, this protein bound to the Schiff base ligand MPM showed the least binding energy (−7.4 Kcal/mol) and single hydrogen bond with ASP-347. The binding energy of the MPM-Cu(II) complex was estimated to be −5.9 Kcal/mol while that of the MPM-Co(II)complex was −6.2 Kcal/mol and the amino acid residues forming the bonds are depicted in Table 3 and Figure 14.

#### 3.5.4. Docking Analysis of *Klebsiella pneumoniae* Protein (3O7J) with Synthesized Compounds

**Figure 15:**
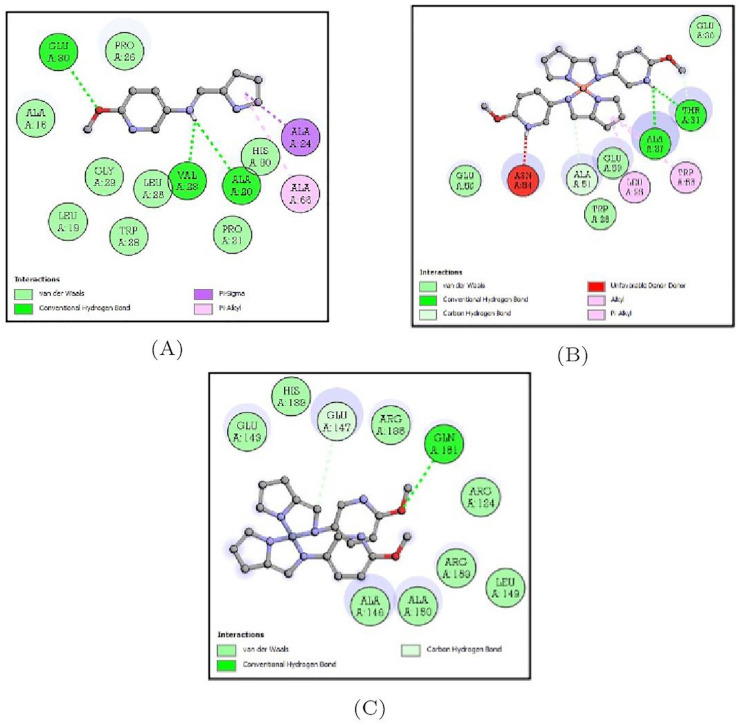
2D representation of the non-bonded interactions between the binding residues of 3O7J protein with (**A**) MPM, (**B**) MPM-Cu(II) and (**C**) MPM-Co(II).

The docking interactions of 3O7J target protein with all three compounds are represented in Figure S12. Of the three compounds, this protein bound to the Schiff base ligand MPM-Cu(II) showed the least binding energy (−6.5 Kcal/mol) and two hydrogen bonds with ALA-27 and THR-31. The binding energy of the MPM ligand was estimated to be −5.4 Kcal/mol while that of the MPM-Co(II) complex was −5.6 Kcal/mol and the amino acid residues forming the bonds are depicted in Table 3 and Figure 15.

### 3.6. Determination of the Global Reactivity Descriptors

The global reactivity descriptors for MPM and its metal complexes have been calculated following the procedure presented in the Materials and Methods Section and are displayed in Table 4:

The electronegativity χ, identified as the negative of the electronic chemical potential (μ), is a measure of the ability of a molecule to attract electrons. In our studied compounds the electronegativity is very similar for the MPM and its complexes, with an interesting result showing that when MPM complexes with Cu (II) the electronegativity is only a bit higher, but for the complex with Co (II) is lower. This can be attributed to the different electronic structures of both ions. This behavior can also observed with the global hardness (η), which is a measure of the ability of the deformation of the electron cloud. A harder molecule will be less reactive than other with a small value of η, resulting in that the MPM-Co(II) will be more reactive than the MPM-Cu(II) complex or than the ligand itself. The electrophiilicity index (ω)i s considered as a better descriptor to provide information on electron transfer and stability. It can be seen from Table 4 that MPM-Co(II) will me a much better electrophile than the other two compounds. As expected from the electronic structure of the studied systems, $\omega^{+}$ will be larger than $\omega^{-}$ for all cases. However, there are again important differences between the values for MPM-Co(II) and the other molecular systems and this also reflected in the values of the net electrophilicity ($\Delta \omega^{\pm }$). This distinct behavior could be the basis for future explanations on the relations between the possible different bioactivity of these molecular systems.

### 3.7. Computational Estimation of the Biological Scores and ADMET Indices

The estimated biological scores are shown in Table 5 while the ADMET indices are reported in Table 6.

It is interesting to observe from Table 5 that the interaction with the different drug receptors will be the same for both complexes. However, it can also be appreciated that with the exception of EI, MPM-Co(II) and MPM-Cu(II) will interact more easily with the different receptors than the ligand. This is translated in the fact that both complexes could be considered as better potential therapeutic drugs than the parent ligand.

In Table 6, we are reporting the computed ADMET properties of the compounds under study. Although the experimental values of these ADMET indices is not known, it can be expected that the calculated values are going to be very reliable because they have obtained through a methodology that has been validated several times in the literature. This information could be of help in the developing research of new therapeutics based on these molecules or related derivatives.

## 4. Discussion

The ligand *N*-((1*H*-pyrrol-2-yl) methylene)-6-methoxypyridin-3-amine has been successfully synthesized with a yield of 78% and used for the synthesis of Cu(II) and Co(II) metal complexes. The proposed structural details of MPM ligand, MPM-Cu (II) and MPM-Co (II) were elucidated by using various analytical tools like Mass spectra, ${}^{1}$H and ${}^{13}$C NMR, FT-IR and thermal analysis. In recent years, transition metal complexes possessing multidentate sorts of different structures have pulled the attention of medicinal chemists to a greater extent because of their fascinating new implementations found in the area of medicine and also in pesticides [73]. Various Schiff based transition metal complexes with oxygen and nitrogen as donor atoms being bidentate exhibits a significant role in the biological framework and may be utilized as models for metallic enzymes that efficiently catalyse the reduction reactions [74]. Moreover, Singh et al. [75] observed that the heterocyclic derivatives of Schiff base have variety of important biological functions, including photosynthesis, oxygen transport in mammalian and other respiratory systems etc., has shown an interesting co-ordination chemistry. In our work, a novel pyridine based methoxy replaced Schiff base ligand and their metal complexes with Cu (II) and Co (II) formation were confirmed by their respective mass spectrum. Further, the signals observed by ${}^{1}$H and ${}^{13}$C NMR spectra confirms the imine (-CH=N) and methoxy (-OCH3) groups present in the synthesized compounds. Metal complexes have recently been discovered to have a huge variety of biological activities, including antimicrobial, antitumor, antifungal, anti-inflammatory and antidepressant activities [76]. According to Mohamed et al. [77] the complexes of 3d transition metal ion exhibit lower toxicity and are capable to penetrate the cell membrane of bacteria when compared to 4d or 5d metal complexes. Thus, the metal complexes of Schiff base considered as a potential therapeutic agent for various diseases is growing fast in the area of bioinorganic science and assist to enhance the nature of the life. But in the present work, the antimicrobial activity study done among the synthesized compounds, it is observed that the synthesized MPM ligand is more potent than its metal complexes. The molecular docking studies carried out for the ligand and its complexes showed low binding energy and hydrogen bond interaction against the receptors. The docking results were compared to similar research [78,79,80] where the authors have carried out antibacterial evaluation of synthesized metal complexes. Souza et al. [78] prepared a copper(II) complex containg thiosemicarbazone ligand and the molecular docking study revealed a structure-activity link between the antibacterial activities and the central copper environments. Similarly, Shridhar et al. [79] synthesized Cu(II) and Co(II) involving the Schiff base of Melonal and concluded that the Co(II) complex exhibited antibacterial activity better than the standard drug amphotericin against *S. aureus* and these findings were supported by molecular docking. Ramashetty et al. *Ramashetty2021* synthesized Co(II) and Cu(II) complexes of the (3-methyl-1-phenyl-4-[(E)-(pyridin-2-yl)diazenyl]-1H-pyrazol-5-ol) ligand and its molecular docking studies showed that these complexes act as potent drugs against the pathogenic organisms *Lebsiella pneumoniae* and *Bacillus subtilis*.

## 5. Conclusions

The newly synthesized Schiff base ligand, MPM and its Cu(II) and Co(II) complexes were structurally confirmed by the analytical and spectroscopic characterization techniques.The antimicrobial activity of the prepared compounds were screened against *Bacillus subtilius*, *Staphyloccocus aureus*, *Escherichia coli* and *Kiebsiella pneumoniae*. The antimicrobial activity of the tested compounds against aforementioned microbial species revealed that the compounds are potent in inhibiting the growth of the microbes. Moreover, the molecular docking studies also revealed that the compounds show low binding energy and good hydrogen bond interaction against the receptors of wo Gram-negative bacteria (*Escherichia coli* and *Kiebsiella pneumoniae*) and two Gram-positive (*Bacillus subtilius* and *Staphyloccocus aureus*).

## Figures and Tables

**Table 1:** Physical data of Schiff base ligand and its metal complexes.

Compound	Molecular Formula	Molecular Mass		Yield (%)
		Calculated	Experimental	
MPM	C_11_H_11_N_3_O	201.22	202.09	78
MPM-Cu(II)	C_22_H_20_CuN_6_O_2_	464.00	463.13	69
MPM-Co(II)	C_22_H_20_CoN_6_O_2_	459.39	458.26	73

**Table 2:** Antimicrobial test results showing the diameter of the zone of inhibition in different concentrations of MPM, MPM-Cu(II) and MPM-Co(II) for respective organisms.

Compound	Concentration (mg/mL)	*B. subtilus* (cm)	*S. aureus* (cm)	*E. coli* (cm)	*K. pneumoniae* (cm)
MPM	25	0.25	0.13	0.56	0.16
	50	1.03	0.37	0.72	0.89
	75	1.28	0.96	1.25	1.72
	100	1.03	1.74	1.80	1.86
MPM-Cu(II)	25	1.74	0.20	0.18	0.11
	50	0.41	0.25	0.92	0.68
	75	0.59	0.74	0.67	1.13
	100	0.72	0.97	1.26	1.63
MPM-Co(II)	25	0.11	0.22	0.28	0.16
	50	0.29	0.64	0.94	0.58
	75	0.28	1.10	0.98	1.72
	100	0.76	1.45	1.58	1.86
Chloramphenicol		2.40	2.54	2.85	3.14

**Table 3:** Molecular Docking analysis of synthesized compounds against bacterial proteins (1QD9, 5C2Z, 5I5H and 3O7J).

SI No.	Bacterial Protein	Compound	Binding Affinity (Kcal/mol)	HB Forming Amino Acid Residues
		MPM	−5.6	-
1	1QD9	MPM-Cu(II)	−6.6	GLY-31
		MPM-Co(II)	−5.5	GLU-117, GLY-31
		MPM	−6.4	HIS-96
2	5C2Z	MPM-Cu(II)	−6.8	GLY-143, ASP-145
		MPM-Co(II)	−7.2	GLY-142, GLY-145
		MPM	−7.4	ASP-268, ASP-347
3	5I5H	MPM-Cu(II)	−5.9	ASN-200
		MPM-Co(II)	−6.2	GLY-142, ASP-145
4	3O7J	MPM	−5.4	GLU-30, VAL-23
				ALA-20
		MPM-Cu(II)	−6.5	THR-31, ALA-37
		MPM-Co(II)	−5.6	GLN-181

**Table 4:** Global reactivity descriptors of MPM and its metal complexes: Electronegativity (χ), Hardness (η), Electrophilicity (ω), EEP ($\omega^{+}$), EAP ($\omega^{-}$) and NE ($\Delta \omega^{\pm }$) (all in eV).

Compound	χ	η	ω	$\omega^{+}$	$\omega^{-}$	$\Delta \omega^{\pm }$
MPM	3.77	3.87	1.84	5.81	2.03	7.84
MPM-Co(II)	3.48	1.48	4.09	10.02	6.54	16.56
MPM-Cu(II)	3.89	3.52	2.15	6.47	2.58	9.05

**Table 5:** Bioactivity scores of the compounds estimated according to the Molinspiration Cheminformatics software for several drug targets of interest for the process of drug discovery.

Compound	GPCR	ICM	KI	NCL	PI	EI
MPM	−0.63	−0.31	−0.15	−0.61	−1.06	0.07
MPM-Co(II)	−0.09	−0.12	0.08	−0.02	−0.19	0.08
MPM-Cu(II)	−0.09	−0.12	0.08	−0.02	−0.19	0.08

**Table 6:** Computed ADMET properties of the compounds according to the admetSAR software.

Ptroperty	Model	MPM	MPM-Co(II)	MPM-Cu(II)
Absorption	HIA	+	+	+
	Caco2	+	+	+
	P-gp Substrate	-	-	-
	P-gp Inhibitor	-	-	-
Distribution	BBB	+	+	+
	CYP450			
	2C9 Substrate	-	-	-
	2D6 Substrate	-	-	-
	3A4 Substrate	-	+	+
Metabolism	1A2 Inhibitor	+	+	+
	2C9 Inhibitor	-	+	+
	2D6 Inhibitor	-	-	-
	C19 Inhibitor	-	+	+
	3A4 Inhibitor	-	-	
Excretion	OCT2 Inhibitor	-	-	-
	AMES Toxicity	+	+	+
	Carcinogens	-	-	-
Toxicity	HERG Inhibitor	-	-	-
	T. Pyriformis Toxicity	-	High	High
	Acute Oral Toxicity	III	III	III

## Data Availability

All data generated from this research is available from the authors under request.

## Supplementary Materials

The following supporting information can be downloaded at online, Figure S1: Mass spectrum of MPM-Cu(II) complex, Figure S2: Mass spectrum of MPM-Co(II) complex, Figure S3: FT-IR spectrum of MPM-Cu(II) complex, Figure S4: FT-IR spectrum of MPM-Co(II) complex, Figure S5: UV-Visible spectrum of MPM-Cu(II) complex, Figure S6: UV-Visible spectrum of MPM-Co(II) complex, Figure S7: TGA of MPM-Cu(II) complex, Figure S8: TGA of MPM-Co(II) complex, Figure S9: 3D visualization of docking analysis of 6HZQ protease binding with (A) MPM, (B) MPM-Cu(II) and (C)MPM-Co(II), Figure S10: 3D visualization of docking analysis of 6D9T protease binding with (A) MPM, (B) MPM-Cu(II) and (C) MPM-Co(II), Figure S11: 3D visualization of docking analysis of 5MVR protease binding with (A) MPM, (B) MPM-Cu(II)and (C) MPM-Co(II), Figure S12: 3D visualization of docking analysis of 3TOT protease binding with (A) MPM, (B) MPM-Cu(II) and (C) MPM-Co(II).
